# Natural language processing for identification of hypertrophic cardiomyopathy patients from cardiac magnetic resonance reports

**DOI:** 10.1186/s12911-022-02017-y

**Published:** 2022-10-18

**Authors:** Nakeya Dewaswala, David Chen, Huzefa Bhopalwala, Vinod C. Kaggal, Sean P. Murphy, J. Martijn Bos, Jeffrey B. Geske, Bernard J. Gersh, Steve R. Ommen, Philip A. Araoz, Michael J. Ackerman, Adelaide M. Arruda-Olson

**Affiliations:** 1 Department of Cardiovascular Medicine, Mayo Clinic Rochester, Rochester, MN USA; 2 Department of Cardiovascular Surgery, Cleveland Clinic, OH Cleveland, USA; 3 Enterprise Technology Services, Shared Service Offices, Mayo Clinic, MN Rochester, USA; 4 Advanced Analytics Services, Mayo Clinic Rochester, Rochester, MN USA; 5 Department of Radiology, Mayo Clinic Rochester, Rochester, MN USA; 6 Department of Pediatric and Adolescent Medicine, Mayo Clinic Rochester, Rochester, MN USA; 7 Department of Molecular Pharmacology and Experimental Therapeutics, Mayo Clinic Rochester, Rochester, MN USA

**Keywords:** Hypertrophic cardiomyopathy, Cardiac magnetic resonance imaging, Natural language processing, Radiology reports

## Abstract

**Background:**

Cardiac magnetic resonance (CMR) imaging is important for diagnosis and risk stratification of hypertrophic cardiomyopathy (HCM) patients. However, collection of information from large numbers of CMR reports by manual review is time-consuming, error-prone and costly. Natural language processing (NLP) is an artificial intelligence method for automated extraction of information from narrative text including text in CMR reports in electronic health records (EHR). Our objective was to assess whether NLP can accurately extract diagnosis of HCM from CMR reports.

**Methods:**

An NLP system with two tiers was developed for information extraction from narrative text in CMR reports; the first tier extracted information regarding HCM diagnosis while the second extracted categorical and numeric concepts for HCM classification. We randomly allocated 200 HCM patients with CMR reports from 2004 to 2018 into training (100 patients with 185 CMR reports) and testing sets (100 patients with 206 reports).

**Results:**

NLP algorithms demonstrated very high performance compared to manual annotation. The algorithm to extract HCM diagnosis had accuracy of 0.99. The accuracy for categorical concepts included HCM morphologic subtype 0.99, systolic anterior motion of the mitral valve 0.96, mitral regurgitation 0.93, left ventricular (LV) obstruction 0.94, location of obstruction 0.92, apical pouch 0.98, LV delayed enhancement 0.93, left atrial enlargement 0.99 and right atrial enlargement 0.98. Accuracy for numeric concepts included maximal LV wall thickness 0.96, LV mass 0.99, LV mass index 0.98, LV ejection fraction 0.98 and right ventricular ejection fraction 0.99.

**Conclusions:**

NLP identified and classified HCM from CMR narrative text reports with very high performance.

**Supplementary Information:**

The online version contains supplementary material available at 10.1186/s12911-022-02017-y.

## Background

Hypertrophic cardiomyopathy (HCM) is the most common inherited cardiomyopathy and a major cause of sudden cardiac death (SCD) in young adults in the United States [1–3]. Cardiac magnetic resonance imaging (CMR) reliably establishes HCM diagnosis and is also important for risk stratification for SCD [3–8]. The interpretation, measurement and phenotypic description of information obtained by CMR exams are routinely reported in radiology CMR reports as narrative text organized in standardized sections in electronic health records (EHRs) [9]. The conversion of narrative text into a computer manageable representation is necessary for extraction of information automatically. This task is accomplished by an artificial intelligence method termed natural language processing (NLP) [10, 11].

It has been established that clinical NLP systems which extract information from radiology reports enable building of patient cohorts, query-based case retrieval and clinical support services [9]. Previous approaches for identification of HCM patient cohorts for research from EHR data have relied upon administrative billing codes [12, 13]. However, information generated clinically (such as CMR results) not relevant from an administrative point-of-view may not be captured by billing codes [10, 14]. No prior reported studies have used rule-based NLP for information extraction of HCM diagnosis from CMR reports. Accordingly, the objective of this study was to assess whether HCM diagnosis can be accurately extracted from CMR narrative reports by rule-based NLP.

## Methods

All methods were performed in accordance with the relevant guidelines and regulations.

### Study design

**Fig. 1:**
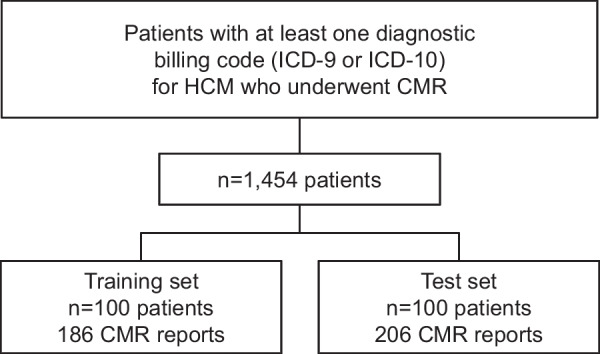
Study design depicting CMR report selection. The study cohort included any patient seen at any Mayo Clinic site between 1998 and 2018 with at least one instance of International Classifications of Diseases 9th revision (ICD-9) or 10th revision (ICD-10) diagnostic codes for HCM. We refined the cohort by specifying subjects in the cohort who had CMR, resulting in a total of 2,051 subjects and 4,934 reports. Of these, 200 subjects were randomly selected and allocated into training and testing sets (100 each). The training and testing sets included 186 and 206 CMR reports, respectively

The study was approved by the Mayo Clinic Institutional Review Board. The subject cohort included any patient seen at any Mayo Clinic practice site from 2004 to 2018 with at least one instance of International Classifications of Diseases 9th revision (ICD-9) or 10th revision (ICD-10) diagnostic codes for HCM (n = 10,015 patients; Fig. 1). Administrative billing codes for HCM diagnosis included I42.1, I42.2, 425.11 and 425.18. The cohort was refined by specifying those who had CMR exams from 2004 to 2008 yielding a total of 1,454 subjects. Of these, 200 subjects were randomly selected and allocated into training and testing sets (100 each). The training and testing sets included 186 and 206 CMR reports, respectively (Fig. 1).

### Manual annotation of CMR reports

**Fig. 2:**
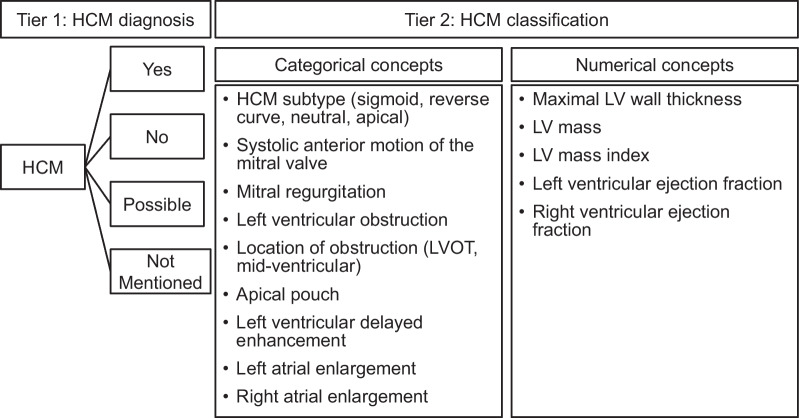
Scheme for CMR report information extraction. We developed NLP algorithms for two objectives: the first, to extract information regarding HCM diagnosis and the second, to extract categorical or numeric concepts for phenotypic classification for reports with diagnosis of HCM by CMR identified by the first-tier algorithm. HCM = hypertrophic cardiomyopathy, LV = left ventricular, LVOT = left ventricular outflow tract

A board-certified cardiologist provided written guidelines which included instructions for manual annotation of CMR reports in the EHR with diagnostic criteria for HCM and examples as well as instructions for abstraction of each of the phenotypic characteristics (Fig. 2). Two trained annotators manually reviewed CMR reports following these written guidelines. CMR reports were categorized into four subgroups based on the presence or absence of CMR diagnosis in the report. Reports diagnostic of HCM were listed as "Yes"; if there was no evidence of HCM or if alternate diagnosis other than HCM was reported, the report was categorized as "No".

Reports interpreted as possible HCM were categorized as "Possible". Reports in which mention of HCM diagnosis was absent were listed as "Not mentioned." Categorical concepts were categorized manually as yes, no, or not mentioned in the report. Values of measurement reported for each numerical concept were abstracted. All reports were reviewed by both annotators; a cardiologist applied standardized criteria to resolve disagreement between annotators thereby creating the gold-standard for comparison.

### Natural language processing

NLP algorithms were developed for two objectives: (1) to extract HCM diagnosis and (2) to extract nine categorical and five numeric concepts for phenotypic classification. The categorical concepts included HCM morphologic subtype, systolic anterior motion of the mitral valve, mitral regurgitation, left ventricular obstruction, location of obstruction [mid-ventricular, left ventricular outflow tract (LVOT)], apical pouch, left ventricular delayed enhancement, left atrial enlargement and right atrial enlargement. Numeric concepts included maximal left ventricular (LV) wall thickness, LV mass, LV mass index, LV ejection fraction and right ventricular ejection fraction.

The scheme for CMR report information extraction by NLP included 15 rule-based NLP algorithms developed to extract phenotypic characteristics from narrative CMR reports (Fig. 2). The rules were developed using MedTagger [15], an open-source NLP tool incorporating dictionary look-up, and regular expression pattern detection which has been used in various clinical NLP applications [15, 16]. MedTagger has been developed and adopted enterprise-wide by Mayo Clinic to deliver NLP services for clinical and translational research and healthcare delivery [17]. MedTagger retrieves lexical variations of user-specified clinical concepts enabled by the Unified Medical Language System Metathesaurus [18]. Given a clinical concept and narrative text, MedTagger generates a table of assertion and negation (present, absent, negated), along with an associated sentence. To improve performance of the base MedTagger rules, additional negations and assertions for each clinical concept were also identified.

### Evaluation and statistical analysis

The performance of each NLP algorithm was compared to gold standard manual annotation of CMR reports. For analysis, reports in the categories HCM “yes” and possible HCM were considered HCM positive whereas reports in the categories “no” and “not mentioned” were considered HCM negative. Performance metrics including accuracy, sensitivity, specificity, negative predictive value (NPV), positive predictive value (PPV) and F1-score were evaluated and calculated as follows: accuracy = (true positives + true negatives)/(true positives + true negatives + false positives + false negatives); PPV = true positives/true positives + false positives; sensitivity = true positives/(true positives + false negatives); NPV = true negatives/(true negatives + false negatives); and specificity = true negatives/(true negatives + false positives); F1 score = 2 × ((PPV × sensitivity)/(PPV + Sensitivity)). Continuous variables were expressed as mean ± standard deviation (SD) or median with interquartile range according to pattern of data distribution. Categorical variables were summarized as counts.

## Results

The training set included 100 subjects (age 57 ± 15 years, 58 men) and the test set 100 subjects (age 56 ± 18 years, 63 men). Examples of phrases extracted from CMR reports by each NLP algorithm are shown in Table 1. In the training set 86 reports were positive for HCM and in the test set 83. The categorical and numerical concepts for HCM classification were extracted from HCM positive reports. Most patients had systolic anterior motion of the mitral valve, mitral regurgitation, LV obstruction and delayed enhancement of the left ventricular walls (Table 2).

**Table 1:** Examples of sentences extracted from CMR reports by NLP

Phenotypic characteristic	Example sentences
HCM diagnosis	*Yes*: Findings *consistent* with *hypertrophic cardiomyopathy* *No*: There is *no evidence for hypertrophic cardiomyopathy*
HCM morphologic subtype	*Sigmoid*: consistent with *sigmoid morphologic subtype of hypertrophic obstructive cardiomyopathy* *Reverse curvature*: consistent with *reverse curve morphologic subtype hypertrophic cardiomyopathy* *Neutral:* Hypertrophic obstructive cardiomyopathy, *neutral subtype* *Apical*: Hypertrophic cardiomyopathy, *apical* morphologic *subtype*
Systolic anterior motion of mitral valve	*Yes: Systolic anterior motion of the mitral valve* is *present* *No: No systolic anterior motion of the mitral valve*
Mitral regurgitation	*Yes*: *Mitral regurgitation* is *present* *No: No mitral regurgitation* seen
Presence of LV obstruction	*Yes:* There is *turbulent flow* in the left ventricular outflow tract *No*: *No turbulence* is seen in the LV outflow tract
Location of obstruction (LVOT, mid-ventricular)	*LVOT:* There is *turbulent flow in the left ventricular outflow tract* *LV cavity: turbulent flow is present in the mid chamber*
Apical pouch	*Yes:* A small *apical pouch is noted* *No: No evidence of apical pouch*
Left ventricular delayed enhancement	*Yes: Delayed myocardial enhancement* within the *left ventricular apex* *No: Delayed myocardial enhancement* is *not present*
Left atrial enlargement	*Yes: Enlarged left atrium* *No: No* significant *left atrial enlargement*
Right atrial enlargement	*Yes: Right atrial enlargement* *No: No* significant *right atrial enlargement*
Maximal LV wall thickness	The *maximal thickness* of the myocardium measured in diastole is *22* mm
LV mass	*LV End Diastolic Mass* = *426* g
LV mass index	*LV End Diastolic Mass Index* = *186* g/m^2^
LV ejection fraction	Hyperdynamic *left ventricular ejection fraction*; *83*%
RV ejection fraction	*RV Ejection Fraction: 61*%

HCM = hypertrophic cardiomyopathy, LV = left ventricular, LVOT = left ventricular outflow tract, RV = right ventricular

**Table 2:** Categorical Information extracted from reports who were NLP HCM positive

Categorical concepts	Training set N of reports = 86		Test set N of reports = 83	
	Present N	Absent/Negated N	Present N	Absent/negated N
HCM morphologic subtype	62	24	62	21
Systolic anterior motion of the mitral valve	55	31	61	22
Mitral regurgitation	57	29	60	23
Presence of LV obstruction	56	30	58	25
Location of obstruction (LVOT, mid-ventricular)	56	30	58	25
Apical pouch	0	86	9	74
LV delayed enhancement	64	22	58	25
Left atrial enlargement	41	45	55	28
Right atrial enlargement	14	72	18	65

HCM = hypertrophic cardiomyopathy, LV = left ventricular, LVOT = left ventricular outflow tract, N = number

The study set included patients with apical morphologic subtype of HCM (training set, n = 10 patients, test set n = 12 patients); neutral septal subtype (training set n = 7 patients, test set n = 2 patients); reverse curve subtype (training set n = 8 patients, test set n = 14 patients) and sigmoid septal subtype (training set n = 37 patients, test set n = 34 patients). When LV obstruction was reported, it was more often located in the LV outflow tract (training set n = 54 patients, test set n = 58 patients) and less likely located in the LV cavity (training set n = 2 patients, test set n = no patients). In both sets HCM patients had increased left ventricular wall thickness and preserved LV ejection fraction (Table 3).

**Table 3:** Numerical information extracted by NLP from reports who were NLP HCM positive

Numerical concepts	Training set N of reports = 86		Test set N of reports = 83	
	N	Mean ± SD	N	Mean ± SD
Maximal LV wall thickness (mm)	78	21.5 ± 4.6	77	20.9 ± 4.5
LV mass (g)	79	197.8 ± 5.5	75	198.2 ± 70.4
LV mass index (g/m^2^)	73	96.8 ± 33.8	71	97.4 ± 30.1
LV ejection fraction (%)	82	72.0 ± 8.0	77	71.4 ± 7.9
RV ejection faction (%)	78	62.7 ± 8.5	75	61.0 ± 7.5

HCM = hypertrophic cardiomyopathy, LV = left ventricular, N = number; RV = right ventricular

**Fig. 3:**
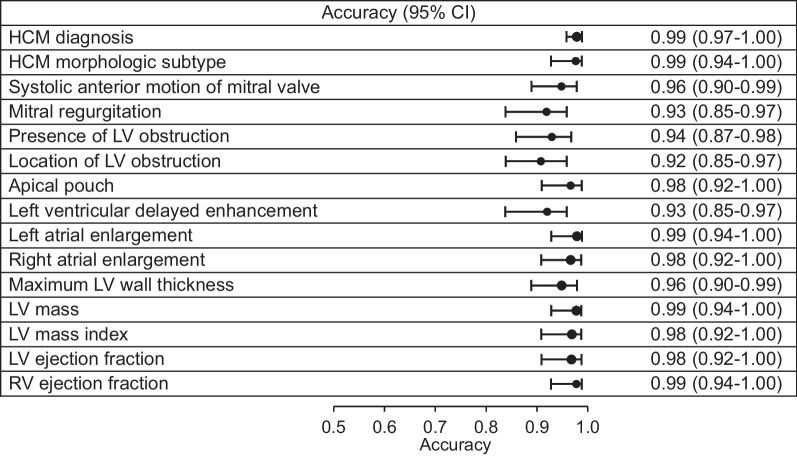
Forest plot summarizing accuracy for extraction of all categorial and numerical variables. The NLP algorithms achieved very high accuracy across all concepts compared to the manually abstracted gold standard. HCM = hypertrophic cardiomyopathy, LV = left ventricular, RV = right ventricular

The NLP algorithms achieved very high performance across all concepts compared to the manually abstracted gold standard (Table 4). NLP had accuracy of 0.99 for extraction of HCM diagnosis from CMR reports. The accuracies for categorical concepts included HCM morphologic subtype 0.99, systolic anterior motion of the mitral valve 0.96, mitral regurgitation 0.93, left ventricular obstruction 0.94, location of obstruction 0.92, apical pouch 0.98, left ventricular delayed enhancement 0.93, left atrial enlargement 0.99 and right atrial enlargement 0.98. One outlier was the performance for extraction of presence of an apical pouch, which had PPV of 0.78 compared to the overall mean of 0.96 for other phenotypic characteristics. It is likely this occurred due to the infrequency of apical pouch in clinical practice. Accuracy for numeric concepts included maximal LV wall thickness 0.96, LV mass 0.99, LV mass index 0.98, LV ejection fraction 0.98 and right ventricular ejection fraction 0.99. Figure 3 shows a forest plot summarizing the accuracies of all categorial and numerical variables**.** Additional performance metrics are displayed in Table 4.

**Table 4:** Performance metrics for each NLP algorithm compared with gold standard

Phenotypic characteristic	Sensitivity	Specificity	PPV	F-1 score	NPV	Accuracy
HCM diagnosis	0.98 (0.92, 1.00)	1.00 (0.97, 1.00)	1.00 (0.96, 1.00)	0.99 (0.97, 1.00)	0.98 (0.94, 1.00)	0.99 0.97, 1.00)
HCM morphologic subtype	0.98 (0.92, 1.00)	1.00 (0.84, 1.00)	1.00 (0.94, 1.00)	0.99 (0.97, 1.00)	0.95 (0.77, 1.00)	0.99 (0.94, 1.00)
Systolic anterior motion of mitral valve	0.97 (0.89, 1.00)	0.95 (0.76, 1.00)	0.98 (0.91, 1.00)	0.98 (0.94, 1.00)	0.91 (0.71, 0.99)	0.96 (0.90, 0.99)
Mitral regurgitation	0.95 (0.87, 0.99)	0.87 (0.66, 0.97)	0.95 (0.97, 0.99)	0.95 (0.91, 0.99)	0.87 (0.66, 0.97)	0.93 (0.85, 0.97)
Presence of LV obstruction	0.94 (0.85, 0.98)	0.95 (0.77, 1.00)	0.98 (0.91, 1.00)	0.96 (0.92, 0.99)	0.84 (0.64, 0.95)	0.94 (0.87, 0.98)
Location of obstruction (LVOT, mid-ventricular)	0.93 (0.85, 0.98)	0.91 0.71, 0.99)	0.96 (0.88, 0.99)	0.95 (0.91, 0.99)	0.84 (0.63, 0.95)	0.92 (0.85, 0.97)
Apical pouch	1.00 (0.59, 1.00)	0.97 (0.91, 1.00)	0.78 (0.40, 0.97)	0.88 (0.60, 1.00)	1.00 (0.95, 1.00)	0.98 (0.92, 1.00)
Left ventricular delayed enhancement	0.93 (0.84, 0.98)	0.92 (0.73, 0.99)	0.97 (0.88, 1.00)	0.95 (0.91, 0.98)	0.85 (0.65, 0.96)	0.93 (0.85, 0.97)
Left atrial enlargement	0.98 (0.91, 1.00)	1.00 (0.88, 1.00)	1.00 (0.94, 1.00)	0.99 (0.97, 1.00)	0.97 (0.82, 1.00)	0.99 (0.94, 1.00)
Right atrial enlargement	0.94 (0.73, 1.00)	0.99 (0.92, 1.00)	0.94 (0.73, 1.00)	0.94 (0.86, 1.00)	0.99 (0.92, 1.00)	0.98 (0.92, 1.00)
Maximal LV wall thickness	0.96 (0.90. 0.99)	1.00 (0.40, 1.00)	1.00 (0.95, 1.00)	0.98 (0.96, 1.00)	0.57 (0.18, 0.90)	0.96 (0.90, 0.99)
LV mass	0.99 (0.93, 1.00)	1.00 (0.59, 1.00)	1.00 (0.95, 1.00)	0.99 (0.98, 1.00)	0.88 (0.47, 1.00)	0.99 (0.94, 1.00)
LV mass index	0.97 (0.91, 1.00)	1.00 (0.69, 1.00)	1.00 (0.95, 1.00)	0.99 (0.96, 1.00)	0.83 (0.52, 0.98)	0.98 (0.92, 1.00)
LV ejection fraction	0.99 (0.93, 1.00)	0.83 (0.36, 1.00)	0.99 (0.93, 1.00)	0.99 (0.97, 1.00)	0.83 (0.36, 1.00)	0.98 (0.92, 1.00)
RV ejection fraction	1.00 (0.95, 1.00)	0.89 (0.52, 1.00)	0.99 (0.93, 1.00)	0.99 (0.98, 1.00)	1.00 (0.63, 1.00)	0.99 (0.94, 1.00)

HCM = hypertrophic cardiomyopathy, LV = left ventricular, LVOT = left ventricular outflow tract, NPV = negative predictive value, PPV = positive predictive value, RV = right ventricular

## Discussion

In this study we describe for the first time novel NLP algorithms for extraction of HCM diagnosis and classification from CMR narrative reports that achieved performance comparable to manual annotation of CMR reports. The results reported herein are important as they suggest that NLP algorithms are sufficiently accurate that they may be deployed not only in research settings but also for potential point-of-care clinical applications.

Narrative text is the most abundant EHR data type and contain as much as 80% of relevant clinical information [10, 11]. In the past, gathering this information has required time-intensive manual review of medical records by providers. However, advances in technology have enabled automated extraction of phenotypic information from narrative notes by NLP. In cardiovascular research, NLP-based systems have been previously used to extract data elements from echocardiography reports, exercise treadmill test reports, and narrative clinical notes on a large scale [16, 19–22]. The study herein developed NLP algorithms which extracted information from CMR reports of HCM patients with high accuracy underscoring the high proportion of true positives and true negatives extracted by NLP compared to the gold standard. The F1 score was also high for most concepts demonstrating low frequencies of false negatives and false positives. One outlier was the lower F1 score for extraction of apical pouch, which likely occurred as a consequence of the low prevalence of apical pouch in clinical practice.

A rule-based 2 tier NLP system for extraction of HCM diagnosis and phenotyping characteristics for HCM classification was developed for this study. Rule-based NLP algorithms have been previously developed for extraction of brain tumor diagnosis and classification [23]. The rule-based NLP approach for extraction of disease diagnosis and classification from narrative reports could be used to develop of NLP algorithms for extraction of disease diagnosis and classification for other cardiovascular diseases and from other types of narrative reports including pathology reports and surgical reports.

We have previously developed a machine learning-based NLP model for HCM classification from radiology reports [24]. The prior model had accuracy between 85–87% in classifying the patients based on HCM diagnosis in radiology reports [24]. The tier 1 of the NLP system described herein had superior performance classifying reports based on HCM diagnosis compared to our prior work. Furthermore, this two tier NLP system also extracted clinically relevant HCM phenotyping characteristics that are necessary for medical management of these patients which will enable implementation of this system in clinical practice via clinical decision support systems.

Given the large volume of EHR narrative reports in contemporary clinical practice, automated methods to assist providers with data extraction, summarization and synthesis have the potential to greatly improve clinical workflow and NLP will be integral to those efforts [10, 11, 14, 25]. The excellent performance of NLP in the study herein suggests potential applications for EHR-based cohort studies and to populate automated point-of-care clinical decision support systems which may be deployed to primary care settings as well as in specialty clinics.

Data from radiology departments are a rich source of information in the form of digital radiology reports and images [26]. Radiology reports are the formal product of a diagnostic imaging referral [9]. A radiology report consists of free text, organized in standard sections which show the diagnosis and information that supports the diagnosis including interpretation, findings and measurements [9]. The review, interpretation and reporting of radiology images are medical procedures performed by trained and licensed radiologists who are physicians with expertise in radiology, which is a medical specialty [27]. The information in radiology reports is used clinically for patient management by other providers with a variety of clinical expertise including primary care, cardiology and surgery.

In clinical practice, providers must find medical information for HCM diagnosis and risk evaluation in radiology reports contained in EHRs which are widely used across the United States [14]. At present, providers are required to gather this information by searching and reading radiology test reports. Providers must then interpret the collected information to make a correct diagnosis and provide a review for their patients at the point-of-care. This provider-review also enables patients to understand their heart condition so they may make informed health decisions in a shared decision-making process. However, the current process for data gathering and summarization of complex medical information can be time-consuming, inefficient, error-prone and may distract providers from interacting with patients during medical encounters.

NLP-enabled clinical decision support tools will allow providers to dedicate more time to patient management, conduct interviews, answer questions and concerns, perform physical examination and assist patients in informed medical decisions instead of spending excessive time searching for information embedded in EHRs required for complex point-of-care discussions and decisions. These computational tools will automatically retrieve and summarize relevant information and display user-friendly synopses at the point-of-care for the benefit of both patient and provider. These tools will also enable health professionals to more promptly and accurately diagnose and manage HCM patients.

The NLP methodology used in the present study for information extraction from clinical narratives contained in radiology reports is different from applications of other artificial intelligence techniques (including deep learning) for extraction of information directly from images which are a separate and promising research field [28, 29]. In the future, information extraction from radiology reports by NLP and imaging processing by other artificial intelligence techniques may complement each other by acquisition of information from different data sources (images vs text in radiology reports) in EHR big data to improve delivery of health care.

Importantly, CMR also identifies phenotypic features of HCM which suggest high-risk of SCD such as extensive delayed myocardial enhancement or extreme hypertrophy [30–32]. In the future, we envision deployment of NLP algorithms to create a dynamic interface to support real-time extraction of HCM diagnosis and phenotypic characteristics from CMR reports which will drive clinical decision support systems to assist providers by displaying relevant information for evaluation and risk stratification of HCM patients which may be automatically input to prognostic models at the point-of-care. Though the phenotypic characteristics extracted were developed specifically for HCM, many can be used for classification of other diseases.

### Limitations

Lessons learned from this study were that complex sentences and ambiguity in language in narrative notes were reasons for incorrect NLP results (see Additional file 1: Table S1). We therefore recommend that interpreting physicians use simple sentences while also avoiding ambiguity of language in creation of reports. Sentences recorded in incorrect sections of the report were also a reason for false-positive results. We suggest text comments appear in the standardized portion of reports. These recommendations may facilitate communication of test results with other providers and improve performance of NLP algorithms for information extraction. The NLP algorithms used were developed and tested in a single tertiary medical center in a cohort of patients with suspected HCM. Future studies should evaluate performance of these algorithms in other medical centers to demonstrate portability.

## Conclusions

NLP identified and classified HCM from CMR narrative text reports with very high performance.

## Supplementary Information

**Additional file 1**. **Supplemental Table 1**. Sample of NLP system errors.

## Data Availability

The datasets generated and/or analyzed during the current study are not publicly available due to participants of this study did not agree for their data to be shared publicly but are available from the corresponding author on reasonable request.

## Abbreviations

HCM: Hypertrophic cardiomyopathy
CMR: Cardiac magnetic resonance imaging
SCD: Sudden cardiac death
EHR: Electronic health record
NLP: Natural language processing
ICD: International Classifications of Diseases
NPV: Negative predictive value
PPV: Positive predictive value
LV: Left ventricle
LVOT: Left ventricular outflow tract
