# Supplementary material for: Natural language processing for identification of hypertrophic cardiomyopathy patients from cardiac magnetic resonance reports
Source: BMC Med Inform Decis Mak. 2022 Oct 18;22:272. doi: 10.1186/s12911-022-02017-y (PMC9580188; doi:10.1186/s12911-022-02017-y)
Supplement: Supplementary file 1 — Additional file 1. Supplemental Table 1. Sample of NLP system errors. [file 12911_2022_2017_MOESM1_ESM.docx]

**Natural Language Processing for Identification of Hypertrophic Cardiomyopathy Patients from Cardiac Magnetic Resonance Reports**

| Nakeya Dewaswala MD ^a*^, David Chen PhD ^b*^, Huzefa Bhopalwala MD ^a^, Vinod Kaggal MS ^c^, Sean P.  Murphy ^d^, J. Martijn Bos MD, PhD ^a^, Jeffrey B. Geske MD ^a^,  Bernard J. Gersh, MB ChB DPhil^a^**,** Steve R. Ommen, MD ^a^, Philip A. Araoz MD ^e^,  Michael J. Ackerman MD PhD ^a, f, g,^ Adelaide M. Arruda-Olson MD PhD ^a^ |  |
| --- | --- |

Department of Cardiovascular Medicine, Mayo Clinic, Rochester, MN, USA ^a^, Department of Cardiovascular Surgery, Cleveland Clinic, Cleveland, OH, USA ^b,^, Enterprise Technology Services, Shared Service Offices, Mayo Clinic, Rochester, MN, USA ^c^, Advanced Analytics Services, Mayo Clinic, Rochester, MN, USA ^d^, Department of Radiology, Mayo Clinic, Rochester, MN, USA ^e^, Department of Pediatric and Adolescent Medicine, Mayo Clinic, Rochester, MN, USA ^f^, Department of Molecular Pharmacology & Experimental Therapeutics, Mayo Clinic, Rochester, MN, USA ^g^.

**Supplemental Table 1.** Sample of NLP system errors.

| **Feature** | **NLP error type** | **NLP Extracted Sentence** | **Missed Sentence** |
| --- | --- | --- | --- |
| **HCM** | Finding in erroneous note section (final diagnosis was incorrectly listed in the section of test type) |  | “MRI of the heart without and with IV contrast for cardiac morphology, function, and delayed enhancement: Hypertrophic obstructive cardiomyopathy with left ventricular hypertrophy.” |
| **Delayed enhancement** | Negation error in complex sentence | “Delayed enhancement imaging showed no evidence for delayed enhancement/infarct.” |  |
| **Systolic anterior motion of mitral valve** | Information extraction error in complex sentence | “There is turbulent flow in the left ventricular outflow tract without systolic anterior motion of the mitral valve.” |  |
| **Systolic anterior motion of mitral valve** | Ambiguity in Note | “No definite SAM but a component of the mitral valve does make contact with the thickened septum during the cardiac cycle likely contributing to obstructive physiology” |  |

**Supplemental Table 1 Legend**: HCM = hypertrophic cardiomyopathy, LVOT = left ventricular outflow tract, NLP = natural language processing, MRI = magnetic resonance imaging; CMR = cardiac magnetic resonance imaging
